# Complete Genome Sequence of Houston Virus, a Newly Discovered Mosquito-Specific Virus Isolated from Culex quinquefasciatus in Mexico

**DOI:** 10.1128/MRA.00808-18

**Published:** 2018-09-13

**Authors:** Nohemi Cigarroa-Toledo, Carlos M. Baak-Baak, Rosa C. Cetina-Trejo, Carlos Cordova-Fletes, Mario A. Martinez-Nuñez, Lourdes G. Talavera-Aguilar, Julian E. Garcia-Rejon, Oswaldo M. Torres-Chable, Gerardo Suzan, Bradley J. Blitvich, Carlos Machain-Williams

**Affiliations:** a Laboratorio de Arbovirología, Centro de Investigaciones Regionales Dr. Hideyo Noguchi, Universidad Autónoma de Yucatán, Mérida, Yucatán, Mexico; b Departamento de Bioquímica, Facultad de Medicina, Universidad Autónoma de Nuevo León, Monterrey, Nuevo León, Mexico; c Laboratorio de Estudios Ecogenómicos, Facultad de Ciencias de la Universidad Nacional Autónoma de México, Mexico City, Mexico; d Laboratorio de Enfermedades Tropicales y Transmitidas por Vector, Universidad Juárez Autónoma de Tabasco, Villahermosa, Tabasco, México; e Departamento de Etología, Fauna Silvestre y Animales de Laboratorio, Facultad de Medicina Veterinaria y Zootecnia, Universidad Nacional Autónoma de México, Mexico City, Mexico; f College of Veterinary Medicine, Iowa State University, Ames, Iowa, USA; Georgia Institute of Technology

## Abstract

We fully sequenced the genome of Houston virus, a recently discovered mosquito-associated virus belonging to the newly established family Mesoniviridae. The isolate was recovered from Culex quinquefasciatus in southern Mexico, which shows that the geographic range of Houston virus is not restricted to the United States in North America.

## ANNOUNCEMENT

Mosquitoes harbor many viruses of medical and veterinary importance, in addition to many mosquito-specific viruses that may suppress or enhance the replication and transmission of pathogenic viruses during coinfection (1). Houston virus is a poorly characterized virus with a presumed mosquito-restricted host range. The virus belongs to the genus Alphamesonivirus in the recently established family Mesoniviridae (2, 3). This study reports the first isolation of Houston virus from Culex quinquefasciatus in southern Mexico and provides a description of the full-genome sequence.

Adult female C. quinquefasciatus specimens were collected in 2017 from houses in the Mayan community of Xkalakdzonot in Yucatan, Mexico. Mosquitoes were collected indoors and outdoors using backpack-mounted aspirators. The collection used for sequencing included 60 female C. quinquefasciatus specimens, which comprised a single pool. The pool was homogenized as previously described (4). An aliquot of the homogenate was inoculated onto subconfluent monolayers of Aedes albopictus (C6/36) cells. Cells were harvested 5 to 15 days postinoculation, and total RNA was extracted using the ZR viral RNA kit (Thermo Fisher Scientific, Waltham, MA). Libraries were prepared from select samples using the TruSeq mRNA stranded kit (Illumina, Inc., San Diego, CA) and analyzed using a NextSeq 500 high-throughput sequencing platform (Illumina). Generated sequence reads were *de novo* assembled using the Trinity software (https://github.com/trinityrnaseq/trinityrnaseq/).

A total of 15,324,522 reads that resulted in 218 contigs were obtained and assembled into a single 20,079-nucleotide (nt) sequence. Sequences recovered from the other mosquitoes will be described elsewhere.

The sequence derived from the pool of C. quinquefasciatus mosquitoes has 99.0% identity to the genome sequences of the four other known isolates of Houston virus, which were recovered from A. albopictus and C. quinquefasciatus specimens collected in the United States (3). Houston virus is a variant of the species Alphamesonivirus 1, which also includes Cavally virus from Cote d’Ivoire, Nam Dinh virus from Vietnam and China, and Ngewotan virus from Indonesia and Australia (5–9). The genome of the isolate identified in our study (designated Hou-Yuc17) shares 91.0 to 98.0% nucleotide identity with the genomes of the aforementioned variants. An inspection of the nucleotide and deduced amino acid sequences of Hou-Yuc17 revealed that the genome contains six major open reading frames (ORFs), consistent with other isolates of Houston virus (3).

To conclude, Houston virus was isolated from mosquitoes in Mexico, and its genome was fully sequenced using Illumina sequencing. This study highlights the fact that the geographic range of Houston virus is not restricted to the United States. Furthermore, this study adds to the sparse number of Alphamesonivirus sequences available in the GenBank database. Future studies should address whether Houston virus impacts the infection, replication, and transmission potential of pathogenic viruses in mosquitoes during coinfection.

### Data availability.

The nucleotide sequence of the Houston virus isolate Hou-Yuc17, identified in this study, has been deposited in GenBank under the accession number MH443059.
